# Gastrointestinal Nematodes among Residents in Melong, Moungo Division, Littoral Region, Cameroon

**DOI:** 10.1155/2021/5368973

**Published:** 2021-02-09

**Authors:** Yamssi Cedric, Noumedem Anangmo Christelle Nadia, Vincent Khan Payne, M. Sabi Bertrand, Ngangnang Ghislain Romeo

**Affiliations:** ^1^Department of Biomedical Sciences, Faculty of Health Sciences, University of Bamenda, P.O. Box 39 Bambili, Cameroon; ^2^Department of Microbiology, Hematology and Immunology Faculty of Medicine and Pharmaceutical Sciences, University of Dschang, P.O. Box 96, Dschang, Cameroon; ^3^Department of Animal Biology, Faculty of Science, University of Dschang, P.O. Box 067, Dschang, Cameroon

## Abstract

**Background:**

Intestinal parasitic infections are among the most common infections worldwide. The present study was undertaken to provide baseline information on the status of gastrointestinal nematodes in Melong Subdivision, Moungo Division, Littoral Region, Cameroon. *Material and Methods*. Seven hundred and eighty-eight stool samples were collected in randomly selected quarters in the community of Melong. These stool samples were brought to the Laboratory of Applied Biology and Ecology in the University of Dschang for analysis using the qualitative (simple flotation) and quantitative (Mc Master count) technique.

**Results:**

The nematodes identified were *Ascaris lumbricoides*, hookworm, *Trichuris trichiura,* and *Capillaria hepatica* with respective prevalences and intensities of infection of 2.2% and 3691.12 ± 3898.47, 1.4% and 940.91 ± 1825.90, 1.0% and 193.75 ± 227.47, and 0.4%and 50 ± 00. The data on the prevalence of nematodes with respect to sex and age showed that females (6.0%) were more infected than males (2.76%) with no significant difference (*P* > 0.05). Furthermore, with respect to age, adults were more infected than children. The percentage of educational level showed a reduction in the number of parasites in the higher educational level. The prevalence of *A. lumbricoides* between localities showed a significant difference (*P* < 0.05) with “Quarter 1” harboring most of the nematodes. Cases of double (*Ascaris lumbricoides* + *Trichuris trichiura*) and triple (*Ascaris lumbricoides* + *Trichuris trichiura* + hookworm) parasitism were encountered with both having a prevalence of 0.3%. According to the fecal concentration of eggs, 63.89% of the infections were light, 5.56% moderate, and 30.56% heavy.

**Conclusion:**

A relatively low overall prevalence was obtained in our study, showing that the national deworming campaign is proving effective, but more effort is needed to completely eradicate these parasites for a single infected individual can cause havoc.

## 1. Background

Gastrointestinal parasites cause considerable mortality and morbidity in the world [1]. These parasites infect both animals and humans [2]. Malnutrition, stunting of linear growth, mental function, verbal ability, physical weakness, and low educational achievement in school children are risk factors associated with gastrointestinal parasites [3]. The severity of the disease on individuals and communities depends on the species of parasite, the nature of the parasite-host interaction, and the nutritional and immunological status of the population [3]. The soil-transmitted helminths (STH) (*Ascaris lumbricoides*, hookworm, and *Trichuris trichiura*) are the most prevalent, infecting an estimated one-sixth of the global population. Infection rates are highest in children living in sub-Saharan Africa (SSA), followed by Asia and then Latin America and the Caribbean (LAC) [4]. According to Brooker et al. [5], the 2006 estimates show that out of the 181 million school-aged children in SSA, almost half (89 million) were infected [5]. The age group which is the most vulnerable to gastrointestinal infections is the children because they rarely employ good sanitary behaviors hence increasing the rate of transmission. [5, 6]. Recent studies have shown the negative effect of soil-transmitted helminth infection on children's school performance [7, 8]. Such infections may weaken the immune system of the host and give way to other nonneglected diseases such as malaria, tuberculosis, and HIV infection [9, 10].

It is estimated that STH affect more than 2 billion people worldwide, and the greatest numbers of infection occur in sub-Saharan Africa, the Americas, China, and East Asia [5, 11]. Ninety-seven percent (97%) of deaths in developing countries are caused by these diseases, and it kills more than 155,000 people per year [11]. According to Essogo [12], out of 16.1 million inhabitants in Cameroon, 10 million are infected with helminths.

The epidemiology of intestinal parasitic infections shows that males and females of all age groups are infected but some age groups are more vulnerable than others [13]. Various studies have shown that the socioeconomic situation of an individual is a very important factor that determines the prevalence of gastrointestinal parasitic infections, having a greater incidence in children [14]. In the years 2002, one or more species of intestinal nematodes infected 250 million people in sub-Saharan African countries according to the World Health Organization. According to Brooker et al. [5], the greatest obstacle to effective control of parasites in at-risk populations is inadequate knowledge of the geographical distribution of infection and the demographic variables that influence the prevalence of infection.

This study is aimed at providing baseline information on the status of gastrointestinal parasites in Melong Subdivision, Moungo Division, Littoral Region, Cameroon.

## 2. Methods

### 2.1. Study Population

Seven hundred and eighty-eight participants from seven different quarters were randomly selected between February and April 2016. This study population was made up of both children and adults with ages ranging between 2 and 81years with a mean age of 22.37 ± 17.9 years. Four hundred and eight of the participants had a level of education (First School Leaving Certificate, Ordinary level, Advanced level, and Bachelor's degree). The study population was made up of 450 (57.1%) females and 388 males (42.9%) with a majority being students.

### 2.2. Sample Size Determination

The sample size of the study population was calculated using the formula *n* = *Z*^2^*α*pq/*α*^2^ as described by Githigia et al. [15]. Where *n* is the required sample size, *Zα* = 1.96 is the standard normal deviation at a 5% level of significance, *p* is the estimated prevalence, and *q* = 1 − *p* and *α* is the precision of the estimate. In a study carried out in the littoral region by Thomas et al. [16], the prevalence of intestinal helminth and protozoa infections in an urban setting of Cameroon: the Case of Douala was 15.2%(p). Our sample size was calculated from this prevalence as follows:

(1) n=Z2αpqα2 =1.96^2×0.1521−0.1520.05^2=198.

Therefore, 198 were the least number of participants to be included in the study.

### 2.3. Inclusion Criteria

1. All those who were at least two years of age
2. All those who signed the informed consent form or those whose parents signed for them

### 2.4. Exclusion Criteria

1. All those who were less than two years old
2. All those who lack capacity, those who did not give their consent, and those who were on anthelminthic treatment within the three previous weeks

### 2.5. Prestudy Visit

The study started with a letter of introduction from the Head of Department of Animal Biology in the University of Dschang to the Regional Delegate of Moungo to obtain a letter of authorization to carry out the research. Copies of this letter of authorization were distributed to quarter heads concerned in churches, in meeting houses, and in all 13 district hospitals of Melong.

### 2.6. Stool Sample Collection and Examination

Proper collection of samples is important for the detection and identification of intestinal parasites. Houses of the quarters concerned were selected at random, and the visit was done from 4 pm because by this time most parents are back from their daily activities. A small screw-capped plastic bottle with a small plastic spoon was provided to each person who fulfilled the above inclusion criteria. They were advised to fill half the bottle with the first faeces first thing the following morning and discard the scoop after use. All the containers were well labeled with the respective sample number, date, and quarter. The participants were advised not to mix the faeces with urine to avoid contamination. The information sheet and informed consent form were written in French and English to ease understanding. The next morning, the information sheet, informed consent form, and specimen bottles were collected. The stool samples were immediately preserved with 10% aqueous formaldehyde solution. After collection, these stool samples were transported in a dark leak proof bag to the Laboratory of Biology and Applied Ecology (LABEA) of the University of Dschang for parasitological analysis. Once in the laboratory, the floatation technique and the Mc Master count technique were used for analysis. People with a positive stool sample were treated free of charge. The intensity of infection was determined from the parasitic load according to the modified classification as shown in Table 1.

### 2.7. Statistical Analysis

The data obtained were stored in a Microsoft Excel spreadsheet and then exported to SPPS (Statistical Package for Social Science, v 21.0) software for analysis. Summary statistics were generated using the same software. The prevalence of parasites was compared between demographic parameters using the chi-square test (*χ*^2^). The Mann–Whitney test was used to compare the parasitic load between sexes while the Kruskal-Wallis test was used to compare the parasitic load between ages. They were all tested at a 5% significance level.

## 3. Results

### 3.1. Characteristic of the Study Population

Seven hundred and eighty-eight participants from seven different quarters were randomly selected between February and April 2016. This study population was made up of both children and adult with age ranging between 2 and 81 years with a mean age of 22.37 ± 17.9 years. Four hundred and eight of the participants had a level of education (First School Leaving Certificate, Ordinary level, Advanced level, and Bachelor's degree). The study population was made up of 450 (57.1%) females and 388 males (42.9%) with a majority being students. The seven quarters that were involved in the study with the number that participated are New Melong (55), Nanci (71), Quarter 2(71), Bonanjo (100), Quarter 1 (137), Houssa quarter (154), and Quartier pont (200).

### 3.2. Overall Prevalence of Nematode Infections

Out of the 788 stool samples examined, 33(4.2%) were infected with at least one parasitic nematode (Table 2). These nematodes included *A. lumbricoides*, hookworm, *T. trichiura*, and *C. hepatica* with specific prevalences of 2.2% (17 infected), 1.40% (11 infected), 1.0% (8 infected), and 0.4% (3 infected), respectively.

### 3.3. The Prevalence of Parasitic Nematodes according to Gender

It can be seen from Table 3 that both sexes were infected by the abovementioned nematodes. Twenty-eight (6.02%) females out of the 450 examined were positive, while out of the 338 males examined, 11 (2.76%) harboured parasites. The overall prevalence in females was greater in all parasites except for *C. hepatica*, that is, *A. lumbricoides* (2.7% females and 1.5% for males), *T. trichiura* (1.3% for females and 0.6% for males), hookworm (2.0% for females and 0.6% for males), and *C. hepatica* (0.02% for females and 0.06% for males). From Table 3, it can be deduced that there is no significant difference between the prevalence of males and females (*P* > 0.05).

### 3.4. The Influence of Age on the Prevalence of Parasitic Nematodes

All age groups were infected with at least one of the nematodes (Table 4). Adults aged from 31 to 40 years had the highest prevalence of infection (*Ascaris lumbricoides* 2.5%, *Trichuris trichiura* 2.5%, and hookworm 3.8%) while the lowest prevalence was recorded in the age group above 50 years with only one parasite (hookworm 2.9%). The prevalence of the parasites had no significant difference between the age groups (*P* > 0.05).

### 3.5. The Prevalence of Parasitic Nematodes according to Level of Education

Four hundred and eight participants had a level of education (First School Leaving Certificate, Ordinary, Advanced, and Bachelor's degree level) (Table 5). Those who had Ordinary Levels were the most infected (7.5%), followed by those with FSLC (4.6%). Only one participant with Advanced level had a parasite while those with Bachelor's degree level were not infected at all. There was no significant difference (*P* > 0.05) between the level of education with all the parasites.

### 3.6. The Prevalence of Parasitic Nematodes according to Locality

Of the 7 quarters involved in our study, at least one parasitic nematode was identified. Quarter 1 showed the highest number of participants infected with *Ascaris lumbricoides* (six cases) and hookworm (four cases), with prevalences of 4.4% and 2.9%, respectively. The prevalence of *Ascaris lumbricoides* had a significant difference between the localities (Table 6).

### 3.7. Prevalence of Single and Mixed Infections

**Figure 1:**
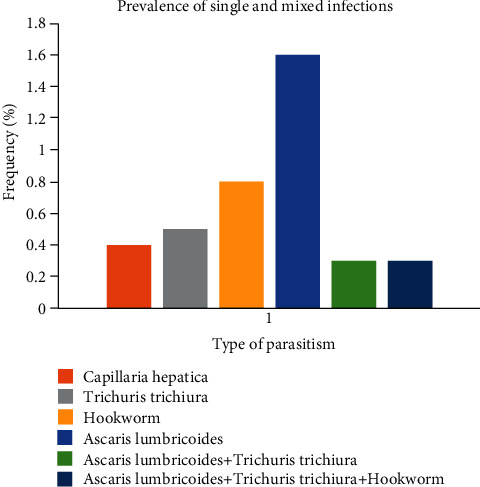
Prevalence of single and mixed parasitic infection.

Figure 1 shows the prevalence of single and mixed parasitic infections. It follows from the analysis of this figure that *Ascaris lumbricoides* recorded the highest prevalence of 1.6% among the single infections. Concerning double infection (*Ascaris lumbricoides* + *Trichuris trichiura*) and triple infections (*Ascaris lumbricoides* + *Trichuris trichiura* + hookworm), both had a prevalence of 0.3%.

### 3.8. Intensity of Infection (Mean EPG) among Residents

The specific intensity of infection which is expressed in terms of the mean concentration of eggs per gram (EPG) of faeces is shown in Table 7. Out of the 788 stool samples examined, *A. lumbricoides* had the highest intensity (3691.12 ± 3898.47) of infection while *Capillaria hepatica* (50 ± 00) had the lowest intensity of infection.

### 3.9. Degree of Infection

In general, 63.89% of infection was light, 5.56 moderate, and 30.56 heavy. All three nematodes (*A. lumbricoides*, *T. trichiura*, and hookworm) recorded light intensity of infection with prevalence 47.1%, 75%, and 81.8%, respectively. Only *T. trichiura* (25%) showed moderate intensity of infection and heavy came from *A. lumbricoides* (52.9%) and hookworm (18.2%) as shown in Table 8.

## 4. Discussion

The present study had a general objective to provide baseline information on the status of gastrointestinal parasites in Melong Subdivision. In this study, the overall prevalence of intestinal nematodes was 33/778 (4.2%), which was lower than in a previous study conducted by Wabo [17] in the Dschang Western Region of Cameroon who had an overall prevalence of 45.3% and in the Western Region (Babadjou) by Payne et al. [18] who had an overall prevalence of 8.5%. In contrast, the prevalence in the present study is very much higher when compared with the study conducted by Zadock [19] in the Same District who recorded an overall prevalence of 0.9%. These differences in prevalence may be attributed to the living standard of the population and the geographical location of the study area [3].

Four parasitic nematodes (*A. lumbricoides*, hookworm, *T. trichiura*, and *C. hepatica*) were identified in our study with specific prevalences of 2.2%, 1.4%, 1.0%, and 0.4%, respectively. These results obtained are lower than those obtained in studies carried out by Wabo et al. [17] with 18% in *A. lumbricoides*, 36% in *T. trichiura*, and 11% in hookworm; [20] who obtained 4.37% in *A. lumbricoides*, 2.7% in *T. trichiura*, and 1.57% in hookworm. *Ascaris lumbricoides* in our study had the highest specific prevalence. This prevalence is lower when compared to studies carried out by Adeyeba and Akinlabi [21] in Nigeria who recorded a prevalence of 46.7%; in the Western Region (Babadjou) by Payne et al. [18] who obtained 4.4%. The high prevalence of *Ascaris* infection could be attributed to the fact that ova of *A. lumbricoides* can survive a prolonged period of 10 years under a warm, shady, and moist environmental condition which could be a reason for their long constant infection [22]. Researchers such as [23, 24] reported that *A. lumbricoides* was much common with ingestion of water and food contaminated with *A. lumbricoides* eggs and occasionally via inhalation of contaminated dust. *Capillaria hepatica* (0.4%) presented the lowest prevalence. This low prevalence of *C. hepatica* (0.4%) may be because it is rarely found in humans, it is a zoonotic disease, and man can only have it from an infected animal [25, 26].

We found in our study that the prevalence of nematode infection was higher in females (6.02%) than in males (2.76%). This agrees with studies carried by Ashrat et al. [27] in the Northern part of the Islamic Republic of Iran in Nour and by Ali [28] in Amol, who had earlier observed that these differences may be related to levels of exposure. Furthermore, this can be explained by environmental factors as more women are occupied in farming [29]. In contrast, studies carried out by Payne et al. [18] in the Western Region Cameroon recorded higher infections in males than in females. This could be attributed to the fact that boys are more often involved in outdoor activities such as playing football and fishing and as such more exposed to infection. The nonsignificant difference (*P* > 0.05) shows the infection is not sex-dependent, meanwhile Wabo et al. [17] observed a significant difference in the prevalence of helminths parasites between both sexes in Cameroon.

We also found out that adults were more infected by these STH than children. All four parasitic nematodes occurred most in adults aged 31-40 years with *A. lumbricoides* and hookworm taking the lead. This agrees with studies carried out by Abelau et al. [30] in Nigeria and by Payne et al. [31] in Cameroon. In contrast, studies carried out in Nigeria by Nmor et al. [32], in the Western Region of Cameroon by Payne et al. [18], in Ethiopia by Gessessew et al. [33] and Mohammed et al. [34] instead showed that children were more infected than adults. The high prevalence in adults than in children could be attributed to the fact that antihelmintic drugs were distributed in schools two months before the start of the study. We also noticed that hookworm infection persisted in old age groups; this agrees with a study carried out by Arene [35] who reported that hookworm infection persists in older adults, especially in farming communities.

The prevalence of intestinal nematodes did not show any significant difference between localities (*P* > 0.05), except that of *A. lumbricoides* which varied significantly (*P* < 0.05) between localities with New Melong harboring most of the parasites. The difference in prevalence may be attributed to hygienic conditions and environmental factors (temperature, relative humidity, and rainfall), which are factors responsible for the geographical distribution of the diseases [36].

Two positive samples with double infections (*A. lumbricoides* + *T. trichiura*) were encountered. Double infections of *A. lumbricoides* and *T. trichiura* had a prevalence of 0.3%. This prevalence is lower when compared with that obtained by Nkengazong et al. [37] who recorded 95.6%, [20] who obtained 1.6% and similar to the 0.5% obtained by Ngangnang et al. [38]. These observations are obvious as the two parasite species are both transmitted by the fecal-oral route (WHO, 2006). Two positive samples also showed triple infection (*A. lumbricoides* + *T. trichiura* + hookworm) with a prevalence of 0.3%. This confirms the triad patterns of *Ascaris*-*Trichuris* and hookworm infections common in most rural communities in Africa [39]. Several studies have shown that multiple infections are more detrimental to the host organism than a single infection [40]. Children having multiple infections have poor academic performance than those of single infections [41].

It is the intensity of infection that is the central determinant of the severity of morbidity [42, 43], but clinical consequences of infection can manifest at much lower worm burdens than previously thought [44]. The intensity of intestinal helminth infections in terms of mean EPG was used in our study. *A. lumbricoides* had the highest mean intensity (3691.12 ± 3898.47) among the nematodes recorded in the study. This is in line with the results of [45]. The mean intensity of *A. lumbricoides* recorded was higher as compared to the results obtained by [18] who obtained 2594.44 ± 3897.174 and slightly lower than that obtained by [17] who registered 3722 ± 5677 for *A. lumbricoides*. This high intensity of *A. lumbricoides* could be due to the fact that *A. lumbricoides* has a higher egg output (200,000 eggs per day) compared to Hookworm and *T. trichiura*.

The categorization of the intensity of infection due to *A. lumbricoides*, *T. trichiura*, hookworm, and *C. hepatica* showed that the majority of infections were light (69.70%) of the positive samples. This is in accordance with [6, 19, 45] who recorded 100% light infection. This may be due to the technique used since the Kato Katz technique is more sensitive than the Mc Master technique used in our study. Furthermore, the classification used for the parasitic load was that of Deuyo [46]. *Ascaris lumbricoides* and hookworm showed heavy infection while *T. trichiura* showed moderate infections. Generally, nematode infections of moderate and high intensity in the gastrointestinal tract produce clinical manifestations. The presence of adult worms in the alimentary canal can cause malabsorption of nutrients and abdominal pains leading to nutritional imbalance and growth failure [6]. The major pathology of hookworm infection results from intestinal blood loss due to adult parasite invasion and attachment to the mucosa and submucosa of the small intestine [11]. Heavy hookworm infection can also lead to chronic protein loss which could result in hypoproteinemia and anasarca [11]. Heavy hookworm infections may be due to the fact that hookworm infection persists in adults [35].

## 5. Conclusion

Our study that had as main objective to determine the prevalence of gastrointestinal nematodes among residents in Melong had an overall prevalence of 4.2%. The four nematodes encountered were *A. lumbricoides*, *T. trichiura*, hookworm, and *C. hepatica*. In the present study, females were more infected than males, and all age groups were infected with at least one of these nematodes with hookworm persisting in old age groups. Adults aged from 31 to 40 years had a trend towards a high prevalence. One type of double (*A. lumbricoides* + *T. trichiura*) and one type of single (*A. lumbricoides* + *T. trichiura* + hookworm) parasitism were identified. *A. lumbricoides* had the highest parasitic mean intensity with the majority of infections being light infections. The study has shown that intestinal nematodes are prevalent in varying magnitude among residents in Melong. The relatively low overall prevalence obtained in our study shows that the national deworming campaigns are proving effective.

## Figures and Tables

**Table 1:** Classification of nematodes according to intensity of infection.

Parasites	Light	Moderate	Heavy
*A. lumbricoides*	50-499	500-999	>1000
*T. trichiura*	50-499	500-999	>1000
Hookworm	50-1049	1050-2000	>2000

**Table 2:** The overall percentage of infected and uninfected individuals.

Parasitic status	No. of individuals	Prevalence (%)
Infected with at least one parasite	33	4.2
Not infected	755	95.8

Total	788	100.0

**Table 3:** Prevalence of parasitic nematodes according to gender.

Parasitic nematodes	Gender			*X* ^2^	Df	*P* value
	Females	Males	Total			
	*N* (%)	*N* (%)	*N* (%)			
*A. lumbricoides*	12 (2.7)	5 (1.5)	17 (2.2)	1.29	1	0.256
Hookworm	9 (2.0)	2 (0.6)	11 (1.4)	2.78	1	0.095
*T. trichiura*	6 (1.3)	2 (0.6)	8 (1.0)	1.056	1	0.304
*C. hepatica*	1 (0.02)	2 (0.06)	3 (0.04)	—	—	—

Total	28 (6.02)	11 (2.76)	39 (4.64)			

**Table 4:** The prevalence of nematode infection according to age group.

Age group (years)	Parasitic nematodes				
	*Ascaris lumbricoidesN* (%)	*HookwormN* (%)	*Trichuris trichiuraN* (%)	*Capillaria hepaticaN* (%)	Total *N* (%)
02-10	6 (2.5)	1 (0.4)	3 (1.2)	2 (0.8)	12 (4.9)
11-20	7 (2.9)	2 (0.8)	1 (0.4)	—	10 (2.9)
21-30	2 (2.5)	1 (1.3)	1 (1.3)	1 (1.3)	5 (6.3)
31-40	2 (2.5)	3 (3.8)	2 (2.5)	—	7 (8.9)
41-50	—	2 (2.6)	1 (1.3)	—	3 (3.9)
>50		2 (2.9)	—	—	2 (2.9)

Total	17 (2.2)	11 (1.4)	8 (1.0)	3 (0.4)	39 (4.95)
*X* ^2^	4.09	7.52	2.61	4.65	
*P* value	0.537	0.184	0.856	0.460	

**Table 5:** The prevalence of nematode infection according to level of education.

Nematodes	Level of education				*X* ^2^	Df	*P* value
	FSLC *N* (%)	Ordinary level *N* (%)	Advanced level *N* (%)	Bachelor's degree *N* (%)			
*A. lumbricoides*	5 (1.7)	4 (5.0)	—	—	3.906	3	0.272
Hookworm	5 (1.7)	1 (1.2)	1 (4.2)	—	0.999	3	0.801
*T. trichiura*	3 (1.0)	1 (1.2)	—	—	0.318	3	0.957
*C. hepatica*	1 (0.3)	—	—	—	0.352	3	0.950

Total	14 (4.6)	6 (7.5)	1 (4.2)				

**Table 6:** Influence of locality on the prevalence of parasites.

Localities	Parasitic nematodes				Total *N* (%)
	*Ascaris lumbricoidesN* (%)	Hookworm *N* (%)	*Trichuris trichiuraN* (%)	*Capillaria hepaticaN* (%)	
Bonanjo	—	1 (1.0)	—	—	1 (1.0)
Haoussa	1 (0.6)	2 (1.3)	2 (2.3)	—	5 (3.2)
Nanci	1 (1.4)	—	—	1 (1.4)	2 (2.8)
New Melong	5 (9.1)	3 (5.5)	1 (1.8)	1 (1.8)	10 (18.2)
Quartier pont	4 (2.0)	1 (0.5)	2 (1.0)	—	7 (3.5)
Quarter 1	6 (4.4)	4 (2.9)	2 (1.5)	1 (0.7)	13 (9.5)
Quarter 2	—	—	1 (1.4)	—	1 (1.4)

Total	17 (2.2)	11 (1.4)	8 (1.0)	3 (0.4)	39 (4.95)
*X* ^2^	21.373	12.194	2.609	7.421	
*P* value	0.002	0.058	0.856	0.284	

**Table 7:** Intensity of infection of nematode parasites.

Nematodes	No infected	Egg per gram (mean ± SD)
*Ascaris lumbricoides*	17	3691.12 ± 3898.47
Hookworm	11	940.91 ± 1825.90
*Trichuris trichiura*	8	193.75 ± 227.47
*Capillaria hepatica*	3	50 ± 00

**Table 8:** Distribution according to fecal concentration of eggs.

Nematodes	Intensity of infection			Total *N* (%)
	Light	Moderate	Heavy	
	*N* (%)	*N* (%)	*N* (%)	
*Ascaris lumbricoides*	8 (47.1)	—	9 (52.9)	17
Hookworm	9 (81.8)	—	2 (18.2)	11
*Trichuris trichiura*	6 (75.0)	2 (25.0)	—	8

*Total*	23 (63.89)	2 (5.56)	11 (30.56)	36

## Data Availability

Data and material are available to other researchers upon request.
